# Relationship Between the *ApaI* (rs7975232), *BsmI* (rs1544410), *FokI* (rs2228570), and *TaqI* (rs731236) Variants in the Vitamin D Receptor Gene and Urolithiasis Susceptibility: An Updated Meta-Analysis and Trial Sequential Analysis

**DOI:** 10.3389/fgene.2020.00234

**Published:** 2020-04-15

**Authors:** Guangyuan Chen, Cong Hu, Yuxuan Song, Mengxi Xiu, Wanfeng Liang, Ningjing Ou, Xiaoqiang Liu, Peng Huang

**Affiliations:** ^1^The Second Clinical Medical School, Nanchang University, Nanchang, China; ^2^Department of Urology, Tianjin Medical University General Hospital, Tianjin, China; ^3^School of Statics and Data Science, Nankai University, Tianjin, China; ^4^Center for Evidence-Based Medicine, School of Public Health, Nanchang University, Nanchang, China; ^5^Jiangxi Province Key Laboratory of Preventive Medicine, School of Public Health, Nanchang University, Nanchang, China

**Keywords:** urolithiasis, variant, vitamin D receptor, meta-analysis, trial sequential analysis

## Abstract

The relationship between urolithiasis and vitamin D receptor (VDR) gene variants is still under debate according to the available published literature. To assess correlations between *VDR* gene variants *ApaI* (rs7975232), *BsmI* (rs1544410), *FokI* (rs2228570), and *TaqI* (rs731236) and urolithiasis susceptibility, we performed the present study through meta-analysis. The PubMed, Cochrane Library, China National Knowledge Infrastructure, EMBASE, Web of Science, and Wanfang databases were searched to retrieve qualified case-control studies. Finally, 31 reports were selected for the present meta-analysis. The results demonstrated that the *VDR* gene *TaqI* TT genotype was related to decreased risk of urolithiasis in the overall population (TT vs. Tt+tt: *P* = 0.011, OR = 0.824, 95% CI = 0.709–0.957). In ethnicity subgroup analysis, we found that the *TaqI* variant was obviously correlated to urolithiasis risk among Asians and Caucasians (*P* < 0.05). Additionally, significant urolithiasis risk was identified in adults. However, the *FokI, BsmI*, and *ApaI* variants did not have an increased risk of developing urolithiasis. Trial sequential analysis results were on a sufficiently large number of participants and did not require more research to confirm associations. Our research suggested that the *VDR* gene variant *TaqI* was correlated with urolithiasis susceptibility and that the t-allele might be the risk gene and T-allele the protective gene in *VDR TaqI* variant.

## Introduction

Urolithiasis is a disease with a prevalence rate of 1 to 5% around the world, and relapse is common after treatment. The 10 year recurrence rate of urolithiasis is up to 50% (Moe and Li, 2018). Epidemiologic research from Europe shows that 4 to 10% of adults get urolithiasis at least one time in their lives, which indicates that urolithiasis is one of the most common diseases threatening human health (Rivers et al., 2000). Causes of urolithiasis are complex and might be related to many factors such as environment, age, habits, genetic factors, metabolic disorders, etc. As more and more mature gene technologies come into use, various genes related to urolithiasis are gradually being discovered.

Vitamin D receptor (*VDR)* belongs to the superfamily of transcription factor nuclear receptors and is a soluble protein that exists in many nuclei and cell membranes (González-Castro et al., 2019). *VDR* is mainly distributed in the kidney, small intestine, skin, and bones. In addition, *VDR* expression is also found in human immune cells (macrophages, monocytes) and many tumor cells (Ou et al., 2014). The high variance of the *VDR* gene is the most important genetic factor that determines the host's ability to respond to the immune system. *VDR* gene variants are thought to be related to susceptibility to urolithiasis. So far, at least 25 *VDR* variants have been found. Among them, *FokI* (rs2228570), *TaqI* (rs731236), *BsmI* (rs1544410), and *ApaI* (rs7975232) are most intensively studied. *VDR gene* is located in 12q13.11 on the chromosome. Among the four *VDR* variants (*FokI, ApaI, TaqI*, and *BsmI*), three of them occur in the intron sections: the *TaqI, ApaI*, and *BsmI* variants, while only the *FokI* variant changes the codon (Ou et al., 2014; González-Castro et al., 2019). On account of the location of the gene, the influence of each variant can be different; for example, *BsmI* and *TaqI* variants can influence the translation efficiency and/or stability of the RNA, but they do not modify the structure of the *VDR* protein (Miyazawa and Suzuki, 2011). Variants of *VDR* have been found to be a significant risk factor for urolithiasis (Rivers et al., 2000; Stamatelou et al., 2003). At present, more and more epidemiologic investigations are centered on the relationship between urolithiasis risk and *VDR* gene variants, but the conclusions remain controversial (Aji et al., 2012; Cakir et al., 2016; Huang et al., 2019). For the above reasons, this meta-analysis was carried out to investigate whether susceptibility to urolithiasis was correlated to the *VDR* gene variants on the basis of widely collected investigations.

## Materials and Methods

### Literature Search

A comprehensive search of the PubMed, Cochrane Library, China National Knowledge Infrastructure, EMBASE, Web of Science, and Wanfang databases and manual search were carried out to find relevant studies. Search strategies were: (*VDR* OR vitamin D receptor) AND (kidney stone disease OR calcium kidney stones OR urolithiasis) AND (variant OR SNP OR genotype OR polymorphism). We searched published studies up to Jan 30, 2020. In addition, we also traced back the relevant references of relevant studies and manually searched relevant articles as well as degree papers.

### Criteria for Inclusion and Exclusion

#### Inclusion Criteria

(1) Case-control studies that focused on the relationship of *VDR* gene variants and urolithiasis; (2) research included *VDR* gene *BsmI, ApaI, TaqI* and *FokI* variants; (3) frequencies of alleles or genotypes in control groups and case groups could be exacted from the articles; (4) the distribution of genotypes of controls were in accord with Hardy-Weinberg equilibrium (HWE).

#### Exclusion Criteria

(1) Reviews, meta-analysis, letters, case reports; (2) studies published repeatedly; (3) results not based on *VDR FokI, BsmI, ApaI*, and *TaqI* variants; (4) works with incomplete data or where data were not available.

### Data Extraction and Literature Quality Evaluation

The Newcastle-Ottawa Scale (NOS) was applied to estimate the quality of relevant articles. If the score was more than or equal to 5 points, the quality of the article was considered to be high. Two people independently extracted data from included documents depending on the formal criteria and then carefully checked the results against each other. If disagreement arose, then the document would be sent to another person for evaluation and review. The data extraction included the first author's name, population ethnicity and area of origin, publication date, sample size of total cases and controls, hypercalciuria in the urolithiasis group, age group, genotype distribution of four *VDR* gene variants, and genotyping methods. The genetic nomenclatures used in this meta-analysis were according to the recommendations of the Human Genome Variation Society (HGVS) and the American College of Medical Genetics and Genomics (ACMGG) (see Table 1).

**Table 1:** Genetic nomenclature used in this meta-analysis according to the recommendations of the Human Genome Variation Society (HGVS) and the American College of Medical Genetics and Genomics (ACMGG).

***VDR* genetic variations**	**Position**	**Genomic description**
*ApaI* (rs7975232)	chr12:47845054 (GRCh38.p12)	NG_008731.1:g.64978G>T
*BsmI* (rs1544410)	chr12:47846052 (GRCh38.p12)	NG_008731.1:g.63980G>T; NG_008731.1:g.63980G>C; NG_008731.1:g.63980G>A
*FokI* (rs2228570)	chr12:47879112 (GRCh38.p12)	NG_008731.1:g.30920T>G; NG_008731.1:g.30920T>C; NG_008731.1:g.30920T>A
*TaqI* (rs731236)	chr12:47844974 (GRCh38.p12)	NG_008731.1:g.65058T>C

### Statistic Analyses

The odds ratio (OR) and 95% confidence interval (95% CI) were used for the analyses. The Cochran's Q-Test and heterogeneity coefficient *I*^2^ were applied to measure the heterogeneity. If *I*^2^ < 50% and *P* > 0.1, it was considered that the studies had no heterogeneity overall, and a fixed-effect model was used; otherwise, a random-effect model was applied. The contrast models were the allele model, dominant model, co-dominant model, homozygote model, and recessive model. Associations of *VDR* variants and urolithiasis under different genetic models were, respectively, analyzed by STATA 12.0 software.

We used Egger's test to evaluate the publication bias. If the *P*-value of Egger's test is more than 0.05 and the funnel plot is symmetrical, it can be considered that there is no significant evidence of publication bias.

We performed a sensitivity analysis to assess whether individual studies affected the overall results. In the end, it was found that after removing each one of the studies in turn, the combined odds ratio (OR) of the remaining studies was within the 95% CI in the meta-analysis. The above results showed that the combined OR of this meta-analysis had good stability.

For subgroup analysis, we divided all subject data into different subgroups so that comparisons could be made between subgroups. Subgroup analysis can be performed on different types of subjects (e.g., different ethnic or age groups). Ethnicity was categorized as Caucasian, Asian, or African. In the present study, we performed subgroup analysis by different ethnicity, age, and calciuria level group.

### Trial Sequential Analysis (TSA)

The TSA was performed using TSA v0.9.5.10 Beta software developed by the Copenhagen Clinical Trial Center in Denmark. This study sets the OR reduction to 20%, the first type of error α = 0.05, and the second type of error β = 0.2 to calculate the required information size (RIS). When the size of the population is accumulating but is less than the expected amount, a trial sequential monitoring boundary (TSMB) is set based on the RIS. We performed this analysis according to the RIS and TSMB. When the cumulative Z-value crosses the TSMB, the results are considered statistically significant. At the same time, it can be considered that the sample size is sufficient.

## Results

### Features of Recruited Studies

**Figure 1:**
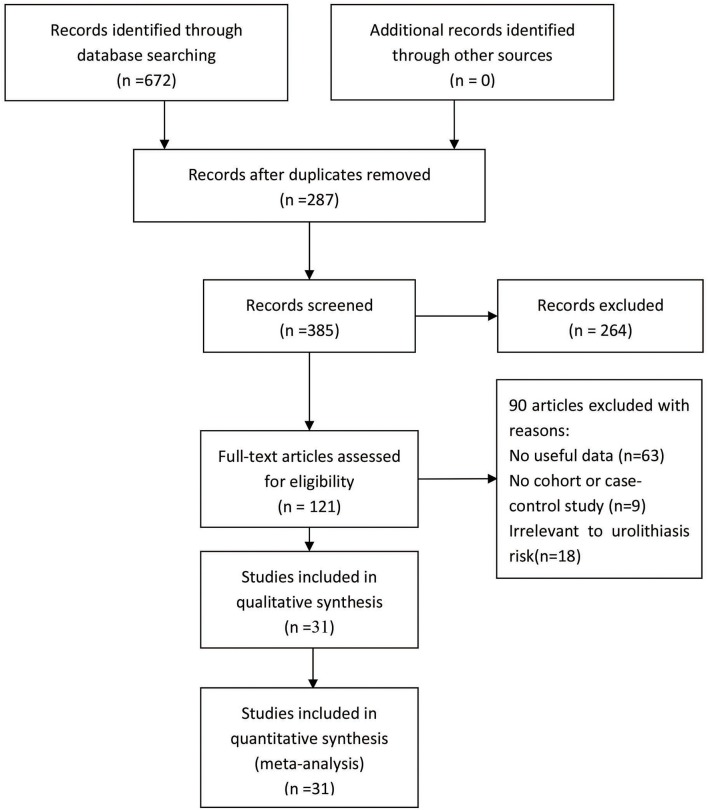
Flowchart illustrating the search strategy for *VDR* variants and the urolithiasis risk.

A total of 31 articles (Jackman et al., 1999; Ruggiero et al., 1999; Chen et al., 2001a,b; Nishijima et al., 2002; Ozkaya et al., 2003; Shaogang et al., 2003; Relan et al., 2004; Rendina et al., 2004; Shao-Qun et al., 2004; Bid et al., 2005a,b; Gunes et al., 2006; Liu et al., 2007; Moyano et al., 2007; Seyhan et al., 2007; Wang et al., 2009, 2012; Li Zhengming and Shi, 2010; Mittal et al., 2010; Mossetti et al., 2010; Seo et al., 2010; Aji et al., 2012; Basiri et al., 2012; Ruan et al., 2012; Guha et al., 2015; Aykan et al., 2016; Cakir et al., 2016; Goknar et al., 2016; Subaşi et al., 2017; Huang et al., 2019) reporting the relationship between *VDR ApaI, BsmI, FokI*, or *TaqI* gene variants and urolithiasis susceptibility were recruited through the search strategy (Figure 1 and Table 2). The data we needed were extracted fully (Table 2). Of these 31 articles, 12 articles studied the BsmI (rs1544410) variant, 19 articles the FokI (rs2228570) variant, 17 articles the TaqI (rs731236) variant, and 13 articles the ApaI (rs7975232) variant. The distribution of genotypes from these 31 studies were all in accordance with HWE.

**Table 2:** Main characteristics of recruited studies.

**References**	**Area**	**Ethnicity**	**Age group**	**Case group of Hypercalciuria**	**Genotyping Methods**	**Case/n**	**Control/n**	***VDR* variants**	**NOS score**
Huang et al. (2019)	China	Asians	Children	NA	PCR-RFLP	130	224	*FokI*	7
Jackman et al. (1999)	USA	Caucasians	NA	Hypercalciuria	PCR-RFLP	17	37	*TaqI*	7
Chen et al. (2001a)	China	Asians	Adults	NA	PCR-RFLP	124	90	*BsmI*	7
Chen et al. (2001b)	China	Asians	Adults	NA	PCR-RFLP	146	90	*FokI*	8
Li Zhengming and Shi (2010)	China	Asians	Adults	NA	PCR- RFLP	50	50	*FokI*	8
Aykan et al. (2016)	Turkey	Caucasians	Adults	NA	PCR-RFLP	164	167	*TaqI*	7
Shaogang et al. (2003)	China	Asians	Adults	Hypercalciuria	PCR-RFLP	150	80	*ApaI, FokI,TaqI*	8
Wang et al. (2009)	China	Asians	Mixed	NA	PCR-RFLP	90	90	*ApaI, FokI*	8
Wang et al. (2012)	China	Asians	Adults	NA	PCR-RFLP	464	450	*ApaI, BsmI,FokI,TaqI*	6
Relan et al. (2004)	India	Asians	Adults	Hypercalciuria	PCR-RFLP	150	100	*BsmI, FokI*	7
Bid et al. (2005a)	India	Asians	Adults	Hypercalciuria	PCR-RFLP	138	166	*FokI*	9
Bid et al. (2005b)	India	Asians	Children	NA	PCR-RFLP	50	60	*FokI*	8
Nishijima et al. (2002)	Japan	Asians	Adults	NA	PCR-RFLP	83	83	*ApaI, TaqI*	8
Shao-Qun et al. (2004)	China	Asians	Adults	Hypercalciuria	PCR-RFLP	186	90	*ApaI, FokI,TaqI*	6
Gunes et al. (2006)	Turkey	Caucasians	Adults	NA	PCR-RFLP	110	150	*ApaI, BsmI,TaqI*	6
Ruggiero et al. (1999)	Italy	Caucasians	Adults	Hypercalciuria	PCR-RFLP	27	150	*BsmI*	7
Liu et al. (2007)	China	Asians	Adults	NA	PCR-RFLP	235	231	*FokI*	6
Mittal et al. (2010)	India	Asians	Adults	NA	PCR-RFLP	125	150	*ApaI, FokI,TaqI*	8
Moyano et al. (2007)	Spain	Caucasians	Adults	NA	PCR-RFLP	51	21	*ApaI, BsmI TaqI*	7
Seyhan et al. (2007)	Turkey	Caucasians	Children	Hypercalciuria	PCR-RFLP	80	40	*TaqI*	8
Ruan et al. (2012)	China	Asians	Adults	NA	PCR-RFLP	169	156	*FokI*	7
Seo et al. (2010)	Korea	Asians	Mixed	NA	PCR-RFLP	102	535	*ApaI, FokI,TaqI*	9
Basiri et al. (2012)	Iran	Caucasians	Adults	NA	PCR-SSCP	106	109	*FokI, TaqI*	7
Cakir et al. (2016)	Turkey	Caucasians	Adults	Hypercalciuria	PCR-RFLP	98	70	*ApaI,BsmI,FokI,TaqI*	9
Ozkaya et al. (2003)	Turkey	Caucasians	Children	Hypercalciuria	PCR- RFLP	64	90	*BsmI,TaqI*	8
Rendina et al. (2004)	Italy	Caucasians	Adults	Hypercalciuria	PCR- RFLP	159	124	*ApaI,BsmI, FokI*	7
Subaşi et al. (2017)	Turkey	Caucasians	Adults	NA	PCR- RFLP	52	51	*BsmI,FokI,TaqI*	8
Aji et al. (2012)	China	Asians	Children	NA	PCR- RFLP	74	103	*ApaI,BsmI*	7
Guha et al. (2015)	India	Asians	Adults	NA	PCR	200	200	*FokI*	9
Rendina et al. (2004)	Italy	Caucasians	Adults	NA	PCR- RFLP	110	127	*FokI*	7
Goknar et al. (2016)	Turkey	Caucasians	Children	Hypercalciuria	PCR- RFLP	78	60	*ApaI,BsmI,TaqI*	8

*NOS, Newcastle-Ottawa Scale; PCR, polymerase chain reaction; RFLP, restriction fragment length variant; SSCP, single-strand conformational polymorphism; NA, not available*.

### Association of Urolithiasis Susceptibility With *VDR TaqI* Variant

**Figure 2:**
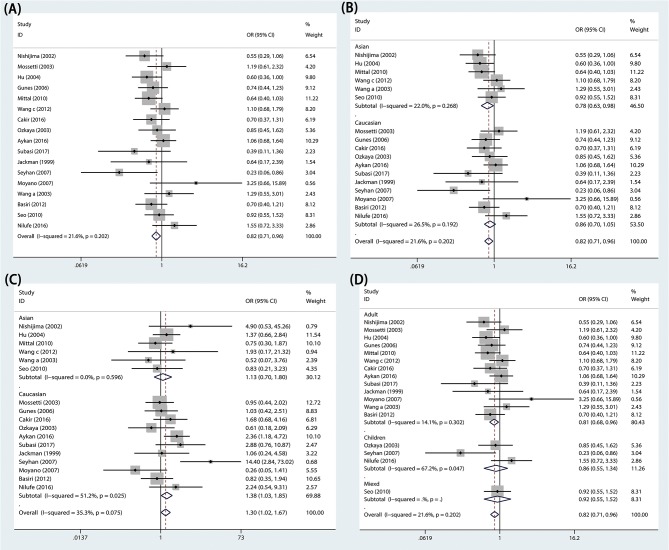
Forest plots of *VDR TaqI* variant and urolithiasis: **(A)** overall for tt vs. TT, **(B)** Asian population for TT vs. Tt+tt, **(C)** Caucasian population for tt vs. TT, and **(D)** adults for TT vs. Tt+tt.

The effects of the *TaqI* variant on urolithiasis susceptibility were investigated in 17 studies, comprising 2,155 cases and 2,326 controls. We found that a significant protective association was observed between urolithiasis susceptibility and the TaqI TT genotype in the overall population (TT vs. Tt+tt: *P* = 0.011, OR = 0.824, 95% CI 0.709–0.957; Figure 2A and Table 3).

**Table 3:** Meta-analysis results.

**SNPs**	**Contrast model**	**Subgroup**	**Number of studies**	**OR (95%Cl)**	***P***	**Test for heterogeneity**		**Analysis model**
						***I^**2**^* (%)**	***P***	
*ApaI*								
	A vs. a	Overall	13	0.951 (0.864, 1.047)	0.305	30.0	0.145	F
	Aa vs. aa	Overall	13	0.877 (0.755,1.018)	0.084	0.0	0.719	F
	AA vs. aa	Overall	13	0.996 (0.807,1.229)	0.970	30.1	0.144	F
	AA vs. Aa+aa	Overall	13	1.010 (0.850,1.199)	0.914	34.4	0.107	F
	aa vs. AA+Aa	Overall	13	1.120 (0.973,1.289)	0.114	0.0	0.503	F
	A vs. a	Asian	8	0.968 (0.813,1.153)	0.715	52.4	0.040	R
	Aa vs. aa	Asian	8	0.864 (0.735,1.016)	0.077	2.9	0.407	F
	AA vs. aa	Asian	8	1.034 (0.693,1.541)	0.871	55.1	0.029	R
	AA vs. Aa+aa	Asian	8	1.011 (0.732,1.398)	0.946	20.8	0.282	R
	aa vs. AA+Aa	Asian	8	1.134 (0.974,1.320)	0.105	33.2	0.163	F
	A vs. a	Caucasian	5	1.002 (0.830,1.211)	0.980	0.0	0.730	F
	Aa vs. aa	Caucasian	5	0.950 (0.647,1.395)	0.793	0.0	0.845	F
	AA vs. aa	Caucasian	5	0.986 (0.652,1.491)	0.946	0.0	0.815	F
	AA vs. Aa+aa	Caucasian	5	1.029 (0.776,1.364)	0.842	20.8	0.282	F
	aa vs. AA+Aa	Caucasian	5	1.040 (0.720,1.503)	0.832	0.0	0.958	F
	aa vs. AA+Aa	Hypercalciuria	5	0.994 (0.745,1.325)	0.966	0.0	0.555	F
	aa vs. AA+Aa	Adult	9	1.127 (0.947,1.342)	0.179	28.2	0.194	F
*BsmI*								
	B vs. b	Overall	12	0.992 (0.876,1.123)	0.900	34.9	0.111	F
	Bb vs. bb	Overall	12	0.911 (0.674,1.232)	0.547	46.2	0.039	R
	BB vs.bb	Overall	12	0.957 (0.734,1.247)	0.745	37.2	0.094	F
	BB vs. Bb+bb	Overall	12	1.061 (0.859,1.311)	0.582	0.0	0.510	F
	bb vs. BB+Bb	Overall	12	1.079 (0.805,1.447)	0.610	50.1	0.024	R
	B vs. b	Asian	3	0.942 (0.745,1.190)	0.617	0.0	0.817	F
	Bb vs. bb	Asian	3	0.942 (0.687,1.292)	0.711	0.0	0.857	F
	BB vs.bb	Asian	3	0.941 (0.544,1.628)	0.828	0.0	0.867	F
	BB vs. Bb+bb	Asian	3	0.943 (0.619,1.437)	0.784	0.0	0.661	F
	bb vs. BB+Bb	Asian	9	1.070 (0.794,1.443)	0.656	0.0	0.980	F
	B vs. b	Caucasian	9	0.990 (0.795,1.231)	0.925	50.7	0.039	R
	Bb vs. bb	Caucasian	9	0.855 (0.552, 1.322)	0.480	60.4	0.010	R
	BB vs.bb	Caucasian	9	0.916 (0.557,1.506)	0.729	53.5	0.028	R
	BB vs. Bb+bb	Caucasian	9	1.104 (0.865,1.410)	0.427	11.1	0.342	F
	bb vs. BB+Bb	Caucasian	9	1.133 (0.737,1.741)	0.570	63.6	0.005	R
	bb vs. BB+Bb	Hypercalciuria	6	1.411 (0.811,2.458)	0.223	69.6	0.006	R
	bb vs. BB+Bb	Adult	10	1.002 (0.761,1.320)	0.988	40.1	0.090	R
*FokI*								
	f vs. F	Overall	19	1.073 (0.897,1.284)	0.440	80.2	*P* < 0.001	R
	Ff vs. FF	Overall	19	1.149 (0.894,1.476)	0.277	75.2	*P* < 0.001	R
	ff vs.FF	Overall	19	1.064 (0.759,1.490)	0.720	68.3	*P* < 0.001	R
	ff vs. Ff+ff	Overall	19	1.013 (0.753,1.361)	0.934	67.4	*P* < 0.001	R
	FF vs. Ff+ff	Overall	19	0.878 (0.679,1.135)	0.320	78.8	*P* < 0.001	R
	f vs. F	Asian	14	1.144 (0.951,1.375)	0.153	77.2	*P* < 0.001	R
	Ff vs. FF	Asian	14	1.174 (0.846,1.628)	0.337	81.9	*P* < 0.001	R
	ff vs.FF	Asian	14	1.200 (0.864,1.666)	0.277	57.2	0.004	R
	ff vs. Ff+ff	Asian	14	1.144 (0.879,1.490)	0.316	50.2	0.016	R
	FF vs. Ff+ff	Asian	14	0.822 (0.596,1.133)	0.231	82.8	*P* < 0.001	R
	f vs. F	Caucasian	5	0.877 (0.522,1.474)	0.621	86.4	*P* < 0.001	R
	Ff vs. FF	Caucasian	5	1.077 (0.820,1.415)	0.594	0.0	0.949	F
	ff vs.FF	Caucasian	5	0.740 (0.263,2.084)	0.569	83.4	*P* < 0.001	R
	ff vs. Ff+ff	Caucasian	5	0.709 (0.255,1.974)	0.510	84.8	*P* < 0.001	R
	FF vs. Ff+ff	Caucasian	5	1.055 (0.722,1.541)	0.782	53.7	0.071	R
	FF vs. Ff+ff	Hypercalciuria	6	0.932 (0.511,1.701)	0.819	85.4	*P* < 0.001	R
	FF vs. Ff+ff	Adult	15	0.861 (0.633, 1.171)	0.340	80.5	*P* < 0.001	R
*TaqI*								
	t vs. T	Overall	17	1.329 (0.956,1.848)	0.091	88.7	*P* < 0.001	R
	Tt vs. TT	Overall	17	1.213 (0.979,1.503)	0.078	38.3	0.055	R
	tt vs.TT	Overall	17	1.289 (0.914,1.817)	0.148	35.3	0.075	R
	tt vs. Tt+TT	Overall	17	1.166 (0.833,1.632)	0.370	48.5	0.013	R
	TT vs. Tt+tt	Overall	17	0.824 (0.709,0.957)	0.011	21.6	0.202	F
	t vs. T	Asian	6	1.648 (0.728,3.729)	0.231	95.1	*P* < 0.001	R
	Tt vs. TT	Asian	6	1.332 (1.055,1.683)	0.016	29.0	0.217	F
	tt vs.TT	Asian	6	1.125 (0.702,1.803)	0.623	0.0	0.596	F
	tt vs. Tt+TT	Asian	6	0.901 (0.574,1.414)	0.650	0.0	0.605	F
	TT vs. Tt+tt	Asian	6	0.782 (0.626,0.977)	0.030	22.0	0.268	F
	t vs. T	Caucasian	11	1.162 (1.013,1.332)	0.032	34.7	0.121	F
	Tt vs. TT	Caucasian	11	1.138 (0.832,1.556)	0.418	43.1	0.063	R
	tt vs.TT	Caucasian	11	1.380 (1.029,1.852)	0.032	51.2	0.025	F
	tt vs. Tt+TT	Caucasian	11	1.271 (0.827,1.952)	0.274	60.8	0.004	R
	TT vs. Tt+tt	Caucasian	11	0.860 (0.702,1.053)	0.144	26.5	0.192	F
	TT vs. Tt+tt	Hypercalciuria	7	0.769 (0.585,1.010)	0.059	33.4	0.173	F
	TT vs. Tt+tt	Adult	13	0.809 (0.684,0.957)	0.013	14.1	0.302	F

*R, random effect model; F, fixed effect model; OR, odds ratio; CI, confidence interval*.

In subgroup analyses, significant urolithiasis risk was identified among Asians (Tt vs. TT: *P* = 0.016, OR = 1.332, 95% CI = 1.055-1.683; TT vs. Tt+tt: *P* = 0.030, OR = 0.782, 95% CI = 0.626–0.977; Figure 2B) and among Caucasians (t vs. T: *P* = 0.032, OR = 1.162, 95% CI = 1.013–1.332; tt vs. TT: *P* = 0.032, OR = 1.380, 95% CI 1.029–1.852; Figure 2C). Furthermore, lower urolithiasis risk was observed among adults (OR = 0.809, 95% CI = 0.684–0.957, *P* = 0.013; Figure 2D). However, patients with hypercalciuria did not exhibit a significant risk of urolithiasis.

### Association of Urolithiasis Susceptibility With *VDR ApaI* Variant

**Figure 3:**
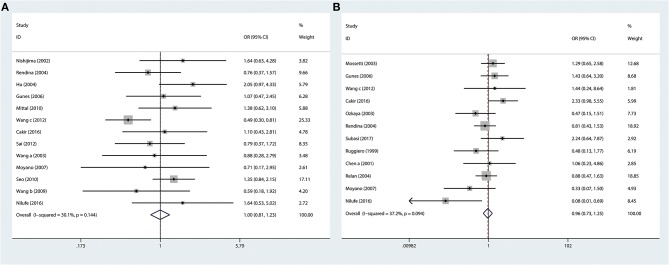
Forest plots of *VDR TaqI* variant and urolithiasis: **(A)** overall for *ApaI* AA vs. aa and **(B)** overall for *BsmI* BB vs. bb.

The *ApaI* variant was investigated in 13 articles. The numbers of cases and controls were 1946 and 2004, respectively. In this meta-analysis, we observed that *VDR ApaI* variant had no association with risk of urolithiasis in the overall population (AA vs. aa: *P* = 0.970, OR = 0.996, 95% CI 0.807–1.229; Figure 3A and Table 3).

In ethnicity subgroup analysis, no relationship was observed among Asians and among Caucasians. In the subgroup of calciuria level, we did not find significant urolithiasis susceptibility among hypercalciuric cases (aa vs. AA+Aa: *P* = 0.966, OR = 0.994, 95% CI 0.745–1.325; Table 3). In subgroup analyses by age, we did not identify any correlation among adult patients (aa vs. AA+Aa: *P* = 0.179, OR = 1.127, 95% CI 0.947–1.342; Table 3).

### Association of Urolithiasis Susceptibility With *VDR BsmI* Variant

Twelve studies (1,481 cases and 1,477 controls) considering the relationship between susceptibility to urolithiasis and *VDR BsmI* variant were selected for this study. In this meta-analysis, we found that *VDR BsmI* variant was not correlated with urolithiasis susceptibility in the overall population (BB vs. bb: *P* = 0.745, OR = 0.957, 95% CI 0.734–1.247; Figure 3B and Table 3).

Among Caucasian and Asian populations, *VDR BsmI* was again not correlated with urolithiasis susceptibility (Caucasian: BB vs. bb: *P* = 0.729, OR = 0.916, 95% CI 0.557–1.506; Asian: BB vs. bb: *P* = 0.828, OR = 0.941, 95% CI 0.544–1.628; Table 3), and neither were there any obvious correlations in subgroup analysis of age group and calciuria level group (Adult: bb vs. BB+Bb: *P* = 0.988, OR = 1.002, 95% CI 0.761–1.320; Hypercalciuria group: bb vs. BB+Bb: *P* = 0.223, OR = 1.411, 95% CI 0.811–2.458; Table 3).

### Association of Urolithiasis Susceptibility With *VDR FokI* Variant

For *VDR FokI* variant, 19 research papers, including 2,847 cases and 2,919 normal controls, were selected. In our meta-analysis, urolithiasis risk had no relationship with *VDR FokI* variant in the overall population (ff vs. FF: *P* = 0.720, OR = 1.064, 95% CI 0.759–1.490; Table 3).

We performed stratification analyses by ethnicity, but no association was observed among Caucasians and Asians (Caucasian: ff vs. FF: *P* = 0.569, OR = 0.740, 95% CI 0.263–2.084; Asian: ff vs. FF: *P* = 0.277, OR = 1.200, 95% CI 0.864–1.666; Table 3). In subgroup analyses by age and calciuria level group, we found no correlation between urolithiasis susceptibility and the *FokI* variant (Adult: FF vs. Ff+ff: *P* = 0.340, OR = 0.861, 95% CI 0.633–1.171; Hypercalciuria group: FF vs. Ff+ff: *P* = 0.819, OR = 0.923, 95% CI 0.511–1.701; Table 3).

### Sensitivity Analyses

**Figure 4:**
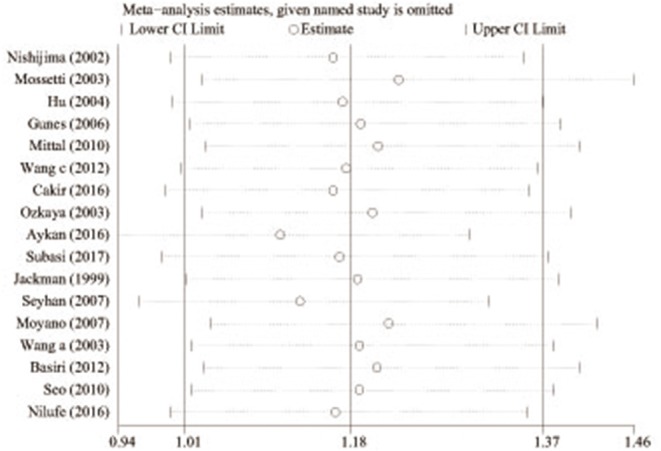
Sensitivity analysis of the pooled ORs and 95% CIs for *TaqI* variant (tt allele vs. TT allele).

Sensitivity analyses were implemented by removing each one investigation from the meta-analysis at a time. Moreover, the pooled ORs did not change markedly [1.01 (Lower Limit) < OR <1.37 (Upper Limit)], which suggested that our results were reliable (Figure 4).

### Publication Bias

Egger's test and Begg's funnel plots were applied to assess publication bias (dominant contrast of pooled analysis: *P* = 0.661 for *ApaI*; *P* = 0.836 for *BsmI*; *P* = 0.953 for *FokI*; *P* = 0.464 for *TaqI*). The final results indicated no publication bias for results relating urolithiasis risk to *VDR* gene variants in the included studies.

### Trial Sequential Analysis Results

**Figure 5:**
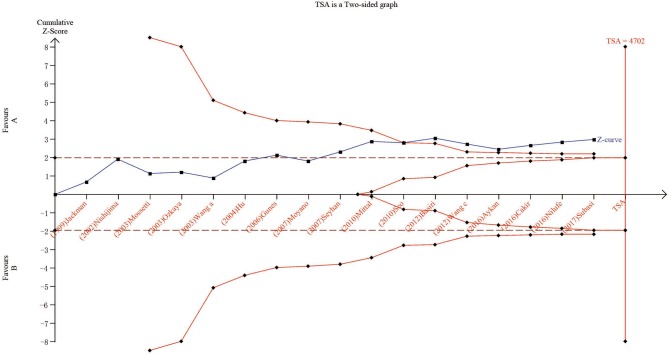
Results of TSA with *TaqI* variant. The required information size was calculated based on a two-sided α = 5%, β = 15% (power 80%), and a relative risk reduction of 20%.

**Figure 6:**
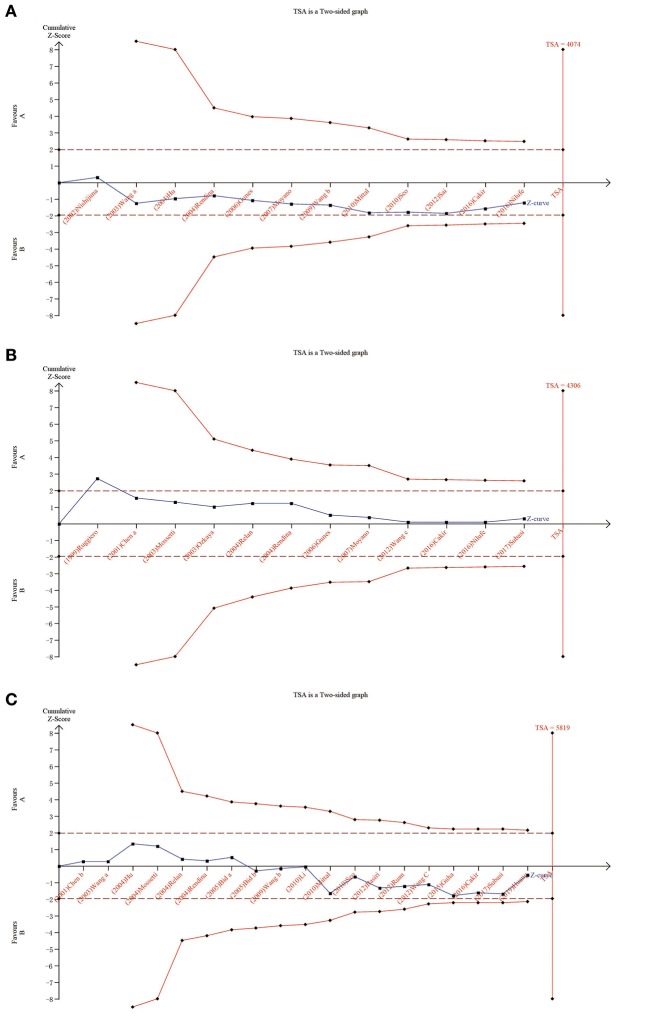
Results of TSA with *BsmI, ApaI*, and *FokI* variants. The required information size was calculated based on a two-sided α = 5%, β = 15% (power 80%), and a relative risk reduction of 20%. **(A)** for *ApaI*; **(B)** for *BsmI*; and **(C)** for *FokI*.

We carried out TSA to reduce the risk of type I error and to assess the RIS. The final results based on the *TaqI* variant suggested that the size of the 11th study crossed the TSA boundary, and the positive conclusion was obtained in advance, which is consistent with the above meta-analysis results, even though the sample size did not reach the required information size (Figure 5; *TaqI*: 4,702 cases). Therefore, it can be asserted that *TaqI* variant TT carriers had a lower risk of urolithiasis than tt carriers and that the evidence was reliable. However, for the *ApaI, BsmI*, and *FokI* variants, the sample size did not reach the required information size (Figure 6A; *BsmI*: 4,306 cases; Figure 6B; *ApaI*: 4,074 cases; Figure 6C; *FokI*: 5,819 cases). Hence, more case-control studies are still needed to confirm the relationship between the *BsmI, ApaI*, and *FokI* variants and urolithiasis susceptibility.

## Discussion

Urolithiasis is a common urological disease. It is affected by a variety of genetic and external factors, including natural factors, social environment, dietary habits, local diseases of the urinary system, and systemic metabolic disorders. Many of these are difficult to control, such as climate, latitude and sunlight, water quality, sex, etc. (Imamverdiev and Guseinzade, 2015). Studies have shown that hot conditions, reduced dietary fiber intake, and high intake of animal protein are important risk factors for urolithiasis (Gouru et al., 2018).

There is a direct link between diet and urolithiasis, and studies have found that excessive intake of high-fat foods has a catalytic effect on the formation of urolithiasis (Shimizu et al., 2012). Because of the high protein content of high-fat foods, it is easy to convert into excreted uric acid, and the appearance of high uric acid can lead to urolithiasis (Lee et al., 2008). Additionally, the amino acids in high-fat foods tend to lead to more obvious urine calcium. Due to a large intake of high-fat food, there will be a large amount of calcium excreted in the urine, which will cause the appearance of urolithiasis (Rotily et al., 2000).

Vitamin D (*VD*) is a fat-soluble substance that is produced due to exposure of the skin to ultraviolet rays from sunlight and is therefore called a “sunshine vitamin” (Philippa et al., 2009). In fact, *VD* is also a steroid hormone in the human body. Normal people can produce *VD* from sunlight exposure when they exercise outdoors. However, some groups of people, such as the white-collar workers, especially those living in the Northern hemisphere, have limited exposure to the sun, and their main source of *VD* is food (Nelson et al., 2015).

*VDR* is a pronuclear protein and belongs to a member of a superfamily that is composed of steroid hormone/thyroid hormone receptors (Ergon et al., 2017). Additionally, it is a nuclear macromolecule that mediates the biological effects of 1,25-(OH) D, which is a type of activated vitamin D substance (Shamran et al., 2017). As we all know, 1,25-(OH) D has a variety of biological functions in our body, such as regulating immune response, controlling cell proliferation and differentiation, and maintaining a mineral balance (Ruggiero et al., 1999). The most important function of 1,25-(OH) D is adjusting the metabolism of phosphorus and calcium in kidney, intestine, and bone, and it is mainly mediated by *VDR* (Ergon et al., 2017; Shamran et al., 2017). In urolithiasis, oxalate, phosphate, and calcium salt are the main components of urinary stones, and 85% are from calcium oxalate (Jurutka et al., 2000). Therefore, urolithiasis is closely related to calcium. As a regulatory gene for the metabolism of calcium, *VDR* is likely to be associated with susceptibility to urolithiasis.

Our meta-analysis systematically investigated correlations between urolithiasis susceptibility and *VDR* variants. For the *TaqI* variant, the TT genotype was significantly associated with decreased urolithiasis risk, while the Tt and tt genotypes elevated urolithiasis susceptibility. Therefore, the t-allele might be the risk gene, and the T-allele might be the protective gene. For the *ApaI, BsmI*, and *FokI* variants, we did not find any associations with urolithiasis risk.

In the age subgroup analysis, we found a decreased risk of susceptibility to urolithiasis associated with the VDR *TaqI* variant. The results suggested that TT genotype carriers were at a lower risk compared to tt genotype and Tt genotype carriers in adults. Thus, the TT genotype might be the proactive factor for urolithiasis susceptibility, while the Tt and tt genotype might be risk factors in urolithiasis.

In terms of the mechanism, the *TaqI* variant does not modify the *VDR* protein structure but can influence the translation efficiency and/or stability of the RNA, which might affect the development of urolithiasis (Jurutka et al., 2000; Uitterlinden et al., 2004). Yamagata et al. (1999) found that in peripheral blood mononuclear cell (PBMC) the *VDR* mRNA levels of allele t were significantly higher than those of allele T. Furthermore, Carling et al. (1998) found that individuals exhibiting the tt genotype had significantly higher *VDR* mRNA levels than those with the TT genotype (Carling et al., 1998). Therefore, it can be considered that people with the t alleles might have increased susceptibility to urolithiasis.

This meta-analysis included 31 case-control studies with 4,144 controls and 3,782 patients, while a previous study by Zhang et al. (2013) only had 23 articles with 2,303 controls and 2,046 cases. Zhang et al. found a decreased risk of urolithiasis associated with the *ApaI* and *TaqI* variants. Nevertheless, we did not find any significant association between urolithiasis susceptibility and the *ApaI* variant. Even in the racial subgroup analysis, there was no significant correlation among the Asian and Caucasian populations. Our meta-analysis included more studies than Zhang's study. Furthermore, in TSA, the cumulative Z-curve crossed the boundary of the TSA, which showed that our meta-analysis had a sufficient number of cases and controls. This may cause the difference between our results and those of Zhang.

Moreover, our meta-analysis also had some advantages. Firstly, our research carried out TSA to analyze the results of the study and the adequacy of the evidence. The TSA results showed that the results of the effect tests on the whole population, Asians, and Caucasians under different genetic models were based on sufficient evidence. Secondly, we carried out subgroup analysis on the basis of a range of related factors. Thirdly, we included more than 30 articles in our meta-analysis. Compared with previous studies, this meta-analysis contained a larger sample size, containing 7,926 subjects, with 3,782 urolithiasis patients and 4,144 non-urolithiasis participants, which was sufficient to draw a reliable conclusion. Fourthly, the subgroup analysis was sufficient. In addition, after Begg's test and sensitivity analysis, the pooled results and conclusions are proved to be credible to evaluate the relationship between urolithiasis risk and *VDR* variants.

Heterogeneity is very important and may affect meta results and result in error. In the present meta-analysis, the contrast model of *ApaI* and *FokI* variants had significant heterogeneity. Thus, we carried out the subgroup analysis of other related factors in order to decrease the sources of heterogeneity. The heterogeneity decreased in subgroup analysis by ethnicity and age.

There were still some limitations to this study that cannot be avoided. First, some unpublished studies with negative results might not be included. Second, this study did not reveal gene–environment and gene–gene interactions due to a lack of enough original information from included studies. Therefore, more multicenter investigations with a large sample are still needed in future to study the association between urolithiasis risk and *VDR* variants.

In conclusion, the results from this systematic review and meta-analysis indicated that urolithiasis susceptibility was associated with the *TaqI* variant. The Tt and tt genotypes could elevate the incidence of urolithiasis, while the TT genotype decreased urolithiasis risk. Therefore, the t-allele might be the risk gene and T-allele the protective gene in the *VDR* gene variant *TaqI*. However, no associations were observed in the *ApaI, BsmI*, and *FokI* variants. Our results also remind us of the necessity of early screening for urolithiasis in *TaqI* variant t-allele carriers.

## Data Availability Statement

The raw data supporting the conclusions of this article will be made available by the authors, without undue reservation, to any qualified researcher.

## Author Contributions

All authors listed have made a substantial, direct and intellectual contribution to the work, and approved it for publication.

### Conflict of Interest

The authors declare that the research was conducted in the absence of any commercial or financial relationships that could be construed as a potential conflict of interest.

## Supplementary Material

Supplementary Table 1 PRISMA Checklist.
